# Controlled root targeted delivery of fertilizer using an ionically crosslinked carboxymethyl cellulose hydrogel matrix

**DOI:** 10.1186/2193-1801-2-318

**Published:** 2013-07-15

**Authors:** Drew W Davidson, Mohit S Verma, Frank X Gu

**Affiliations:** Department of Chemical Engineering and Waterloo Institute for Nanotechnology, University of Waterloo, 200 University Avenue West, Waterloo, Ontario N2L 3G1 Canada

**Keywords:** Carboxymethyl cellulose, Controlled release, Fertilizer, Agrochemicals, Wheat

## Abstract

**Aims:**

The recent increases in food prices caused by the corresponding increases in fertilizer costs have highlighted the demand for reducing the overuse of fertilizers in industrial agriculture. There has been increasing interest in developing plant root-targeted delivery (RTD) of fertilizers in order to address the problem of inefficient fertilizer use. The aim of this study is to develop a low cost controlled release device to deliver fertilizers to plant roots and thereby increase fertilizer use efficiency.

**Methods:**

The Root Targeted Delivery Vehicle (RTDV) is formed by dissolving Carboxymethyl Cellulose (CMC) chains in water, mixing it with liquid fertilizer and crosslinking using iron and calcium salts. Basic measurements quantifying nutrient release and green house growth trials were carried out to evaluate fertilizer use efficiency on wheat growing in nutrient depleted soil media.

**Results:**

Growing wheat in nutrient depleted media showed that the RTDV permits a 78% reduction in the amount of fertilizer needed to achieve similar levels of plant yield in these conditions. Quantifying the losses associated with the RTDV synthesis showed that optimizing manufacturing could possibly increase this value as high as 94%. Furthermore, the delivery device showed a similar lifetime in soil to the plant’s growth cycle, delivering fertilizer over the course of the plant’s growth before removal from soil by degradation.

**Conclusions:**

These results illustrate the importance of fertilizer delivery in facilitating absorption and may have potential to vastly increase the use efficiency of fertilizers in soil, resulting in a significant reduction of costs and environmental damage. With more in depth study to quantify the fertilizer release and refine the device, there is great potential for the use of the RTDV as an effective means to increase fertilizer use efficiency in agriculture.

## Background

Fertilizer usage has a critical impact on all aspects of agriculture, either directly or indirectly, by altering the cost of growing and the relative profitability of crops. Use of Controlled Release Fertilizers (CRF) allows the release of nutrients to be better matched with the life cycle of the plant (Malhi et al. 2010 Shaviv and Mikkelsen 1993) in order to increase the efficiency of nutrient uptake by plants. Furthermore, the nutrient demands of the plant can be met more closely by designing an appropriate controlled release system to increase efficiency and reducing the risk of overdosing the plant (Akiyama et al. 2010). CRF also prevent fertilizers from being leached from the soil and decrease costs for agriculture by reducing the amount of fertilizer needed and the labour and fuel costs associated with repeated applications of fertilizers (Shaviv and Mikkelsen 1993Akiyama et al. 2010Simonne and Hutchinson 2005). There is also evidence that different nutrients and micronutrients can influence the ability of plants to utilize other nutrients effectively (Shaviv and Mikkelsen 1993Garcia-Mina et al. 2004).

Polymer coating of large fertilizer granules is the most commonly applied controlled release mechanism and it relies on the biodegradation of the polymer coating to release fertilizers over the course of a growing season. Coating the fertilizers with a polymer serves to help immobilize the fertilizer pellets and hence make them resistant to runoff and leaching (Morita et al. 2002); varying degrees of leaching resistance have been observed depending on the type of CRF used. The encapsulation also protects fertilizers from environmental degradation from microorganisms or chemical reactions and lengthens the amount of time plants have to absorb the fertilizer (Tian and Saigusa 2005). However, the commercially available CRF are vulnerable to changes in the soil types, moisture contents and other factors which can affect the release rate (Chen et al. 2008). These complications can lead to fertilizer release not being synchronized with plant demand and may create situations where the plants are starved of nutrients or unable to use the fertilizer released (Gumbo et al. 2008).

We hypothesize that a Root Targeted Delivery Vehicle (RTDV) fabricated from Carboxymethyl Cellulose (CMC) hydrogel is able to decrease the amount of fertilizer necessary for maintaining plant yields. The efficiency of fertilizer use by the plant would be increased by delivering fertilizer to the plant at a rate that neither allows the fertilizer to be lost nor causes the plant to be undernourished. CMC material is able to fill the role of a CRF due to its low cost, due to its common use as filler in food products, and its ability to directly encapsulate fertilizers. Unlike other hydrogel materials being developed for fertilizer release, the CMC hydrogels detailed in this report do not coat granules of fertilizer with a layer of hydrogel like commercial fertilizers and some other hydrogel based devices (Ni et al. 2011) but instead the hydrogel matrix is mixed with liquid fertilizer. Intercalation of the fertilizer greatly simplifies the synthesis of the CRF by removing the need for steps such as milling and filtering and coating processes such as fluidized bed used in the manufacture of other emerging hydrogel CRF products (Ni et al. 2011Zhao et al. 2010). Furthermore, since the fertilizer is contained within the hydrogel material rather than encapsulated, the hydrogel is not vulnerable to physical damage or any other process which can rupture a coating and cause device failure. The hydrogels can be dried in order to allow the hydrogel material to be stored and mixed into soil, which gives the material the potential to be used for deployment on a large scale.

In addition, this hydrogel device would also be able to serve as a platform for the release of multiple compounds for root targeted delivery. This control over fertilizer release is possible due to CMC hydrogels being able to encapsulate a wide variety of bioactive molecules, both hydrophobic and hydrophilic, such as glucose oxidase, aldicarb (a carbamate insecticide), and bovine serum albumin (Wu and Choi 2004Kok et al. 1999Zhang et al. 2001Kulterer et al. 2012). Even microorganisms such as *Pannonibacter phragmitetus* may be encapsulated by CMC materials (Xu et al. 2011).

The RTDV attempts to utilize the principles of controlled drug release to maximize the absorption and utilization of the released fertilizer by the wheat plants. This strategy involves optimizing the rate of release of the RTDVs while simultaneously targeting the delivery to plant roots in order to minimize fertilizer losses to runoff and chemical degradation. Using this design of CRF, it was demonstrated that plant growth can be increased and fertilizer use can be significantly decreased without a significant decrease in plant yield. The potential for the RTDV for controlled release of hydrophilic and hydrophobic materials for applications such as pest and disease control will also be demonstrated. The results of the study also demonstrate the potential for the RTDV to be simultaneously used to increase drought resistance of crops by improving soil water retention.

The CRF system currently being designed has the ability to deliver both major nutrients such as nitrogen, phosphorous and potassium compounds as well as small molecules such as micronutrients. With further research the release of these nutrients will be targeted to the root zone and lifecycle of the plant to maximize the usage efficiency of the plant in order to reduce fertilizer related costs. In addition to the reduction of costs by increasing the efficiency of fertilizer use, CRF serve to reduce the environmental pollution caused by fertilizer application (Gabriels et al. 2001Gumbo et al. 2008Anderson 2009).

## Materials and methods

### Materials

CMC powder (MW 250000), iron (II) chloride pentahydrate, iron (III) chloride hexahydrate were used as received from Sigma Aldrich. Anhydrous calcium chloride and regenerated cellulose membrane (nominal MWCO 12000–14000, width 45 mm) were used as received from Fischer Scientific. 20-20-20 fertilizer was obtained from Plant Products Co. Turface used as a nutrient depleted growth media was obtained from Profile Products. Organic wheat seeds were obtained from Eating Well Organically.

### RTDV synthesis

The RTDVs are manufactured through dissolution of 7 g CMC powder in 100 ml deionized (DI) water. The gel is then loaded with the desired release formulation, in this case the 20-20-20 fertilizer, by dissolving the desired amount of fertilizer in 10 ml deionized water then mixing the dissolved fertilizer into the hydrogel. After loading, the gel is placed into a 30 ml syringe and injected into sections of dialysis membrane. The membrane is then clamped at both ends and left in an ionic crosslinking solution composed of 40 g calcium chloride, 4 g iron (II) chloride and 4 g iron (III) chloride dissolved in 400 ml DI water for 48 hours in order to obtain the resulting ionically crosslinked hydrogel. Afterward, the crosslinked hydrogels are taken out of solution and the membrane is removed. Drying of the RTDVs involves placing the RTDVs into an oven set at 80°C for 24 to 48 hours depending on the desired level of crystallization for the hydrogel.

### Release study

Bench-top release studies were performed by immersing the fertilizer loaded RTDVs into 150 ml DI water. Three 300 μl samples were taken on a regular basis and analysed using UV–vis spectrometry, described below. Volumes of release media removed for sampling were replaced with DI water. Release media was replaced daily with fresh DI water to avoid saturation of the media. The study continued until all fertilizer was released from the gels as noted by steady readings for the UV–vis measurements. The combination of conditions used for synthesis of RTDVs is summarized in Table 1. Here, full dose represents the total mass of fertilizer (2.15 g) used for the positive control experiments given daily applications of liquid fertilizer. The fertilizer loading (wt%) is calculated by dividing the mass of fertilizer by the mass of polymer used.

**Table 1:** **Synthesis conditions of root targeted delivery vehicles (RTDV)**

Sample	Fertilizer loading (% of full dose)	Fertilizer loading (wt%)	Drying (hours)
RTDV 1 ^a^	22	20	0
RTDV 2 ^a^	33	30	0
RTDV 3 ^a^	44	40	0
RTDV 4 ^a^	22	20	24
RTDV 5 ^a^	22	20	48
RTDV 6 ^a^	100	92	0
RTDV 7 ^a^	100	92	24
RTDV 8 ^a^	100	92	48
RTDV 9 ^a^	44	40	24
RTDV 10 ^b^	44	40	0
RTDV 11 ^c^	44	40	0

^a^ refers to devices synthesized with the method detailed in *RTDV synthesis* above, ^b^ refers to devices synthesized while heating the crosslinking solution to 40°C as detailed in *alternative RTDV synthesis methods* below, ^c^ refers to devices synthesized using elevated levels of iron salts as detailed in *alternative RTDV synthesis methods*.

### UV–vis measurements

UV–vis measurements were done to measure the absorbance of fertilizer in solution in order to determine the concentration. Readings were taken using a Bio-Tek Epoch microplate spectrophotometer on 300 μl samples. Absorbance readings were performed at 630 nm, which corresponds to an absorbance peak for the 20-20-20 fertilizer compound. Measurements were done in triplicates to account for experimental error. This method was employed because it was simple and rapid. The linear dependence of absorbance on the concentration of the fertilizer was verified by obtaining a calibration curve. Although this method does not measure all the nutrients that are being released, it provides an indication of controlled release of fertilizer which is further verified by the greenhouse experiments. The UV–vis measurements are able to indicate the overall rate of release from the device but are not able to quantify the concentration of individual fertilizer components. Although it would be preferable to obtain this information, it was deemed to be beyond the scope of this initial study. More in- depth quantification of the release of fertilizer from the RTDV is planned in the future.

### Growth testing

Growth testing using plant models was done in a temperature and humidity controlled greenhouse. The testing involved placing the CMC hydrogels prepared using the above method into a small planter filled with Turface. The gel is covered with Turface and a wheat seed is placed into the pot and again covered with Turface. The pots are then watered to hydrate the soil. The growth experiments involving wheat continue for 50 days with 50 ml deionized water applied daily to allow the wheat to reach the heading stage, which occurs at approximately 45 to 50 days for the wheat strain used. The heights of the plants are recorded throughout the experiment in order to gauge the hydrogel’s performance in increasing plant growth. At the end of the experiment the plants are removed from the soil and are dried completely in ambient conditions in order to obtain dry mass measurements. Each growth experiment is performed alongside a negative and positive control to help minimize the effect of variation from the experiments occurring at different times. Positive controls received 50 ml deionized water for the first 7 days and 1 g/L 20-20-20 fertilizer dissolved in 50 ml deionized water per day for the remaining 43 days instead of using a hydrogel device for fertilizer delivery. Negative controls received only 50 ml deionized water and did not use a hydrogel device either. All experiments are done in replicates of three to account for variation between plants. Plants that do not germinate within the first 20 days are considered to be outliers and their measurements were not considered in the final average.

## Results and discussion

### RTDV synthesis and release testing

The controlled release device is made through ionic gelation of CMC using iron and calcium salts. Raw fertilizer containing nitrogen, phosphorous and potassium was added to the CMC immediately prior to gelation. Free fertilizer and salts were removed from the hydrogel by washing with deionized water. The hydrogels were synthesized in 5 cm × 3 cm × 1 cm pieces in order to maximize the compatibility of the gels with the growth experiment set-up and for ease of manufacture using the ionic bath. This size of hydrogel was also necessary for allowing the hydrogels to be able to hold enough fertilizer for the duration of the growth experiment. The hydrogels were tested at three different loading levels, 22%, 44% and 100% dosage, as compared to the total amount given to plants that were administered daily applications of fertilizer.

**Figure 1:**
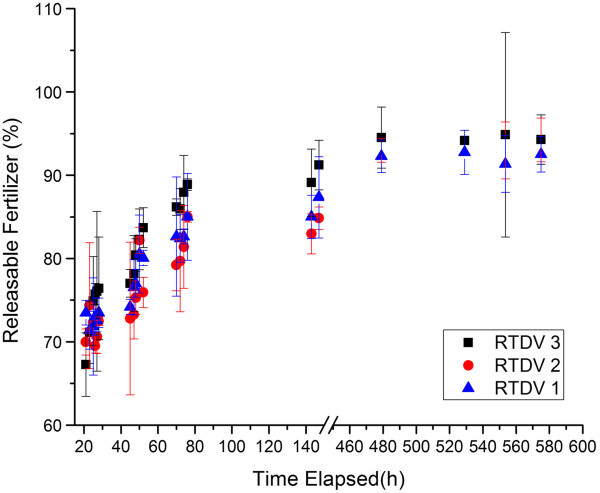
**Fertilizer release profiles of RTDVs in deionized water.** The hydrogels were loaded with 20-20-20 fertilizer at 22% (“blue triangle symbol” RTDV 1), 33% (“red circle symbol” RTDV 2) and 44% (“black square symbol” RTDV 3) dose levels. The amount of fertilizer released (mean ± S.D., n = 3) was quantified using UV-Visible spectrophotometry.

In order to determine the optimal loading levels for the ionically crosslinked RTDV, measurements of the release rates of several different hydrogels were taken in order to quantify the effects of increasing the fertilizer loading level in the RTDV. The results showed that as the fertilizer concentration increased, the initial burst release of fertilizer increased and the release was less sustained than with lower fertilizer loading levels. The RTDVs released 80 to 90% of the loaded fertilizer within the first 80 hours. However, after the initial release, there was a steady release of fertilizer until after 500 hours, after which the release ceased. The efficiency of release, the total amount of fertilizer released from the RTDVs compared to the amount loaded, is on average 96%, indicating that the RTDVs are able to release the majority of their payload with minimal irreversible binding. On the basis of the release profiles of the RTDVs at different loadings (Figure 1), 22% dose and 44% dose loadings were chosen for testing since 22% dose shows the strongest sustained release of fertilizer over the long term, while the 44% dose device had a similar release profile to the 33% dose device but with a higher loading level.

The ionic crosslinking process of the RTDV relies on the interaction of the carboxyl group of the CMC with polyvalent salts (Rathna et al. 1996) in order to create a more stable hydrogel than that which is obtained naturally by the dissolution of CMC in water. The objective of the study is to create a controlled release device capable of increasing the fertilizer use efficiency of plants. However, the 20-20-20 fertilizer used in the experiment has a number of ionic compounds which weaken the overall integrity of the hydrogel. At high concentrations, the presence of chelators in the fertilizer weakens the bond strength of the CMC crosslinks (Rathna et al. 1996). Thus, the RTDVs loaded with lower fertilizer levels are expected to sustain release for a longer period of time.

Natural soils were avoided in this experiment to prevent confounding of results. A nutrient depleted medium assures that the growth was due to the implanted RTDV instead of nutrients from the soil itself. The release study indicated a burst release by the hydrogel in the first 24 hours in advance of the RTDV’s sustained release period. Turface was chosen as the soil media for the growth experiments in order to compensate for any possible burst release by the RTDV by preventing the build-up of harmful levels of nutrients in the soil. Typically, plants gain little benefit from soil nutrients before seed germination so this burst release was expected to have little impact on the study, as the coarse grain of the soil media would not retain the fertilizer effectively, preventing excessive nutrient concentrations that may harm the plant. Thus, the growth experiments give a better representation of the effects of the sustained release fertilizer due to the nutrient depleted soil matrix. In addition, the presence of the burst release coupled with the low retention of the soil indicates that plants are likely receiving only a fraction of the fertilizer loaded into the RTDV. In order to prevent this excess fertilizer from being wasted an extra processing step where the RTDV was washed in deionized water before implanting in soil, removing the loosely bound fertilizer from the device and making them available for recycling, is planned to be added to the device synthesis.

The sustained release from the RTDV in water in the release study is still somewhat rapid, however, other water and soil based release studies done using CMC (Kumar et al. 2010) have shown that release of hydrophobic compounds in soil using CMC based controlled release media can be nearly twice as slow as in water (Kumar et al. 2010). Considering that the fertilizer being released from the RTDVs is hydrophilic and observations from growth testing showing fertilizer remaining in the RTDVs even after 50 days, it is likely that the release of fertilizer in soil will be much more prolonged compared to the timing given by the water based release experiments.

An alternate setup using fertilizer pellets coated with a different form of CMC hydrogel showed that their formulation reached a release of 80% after approximately 5 days in soil (Ni et al. 2011). Due to the differences in the release study procedure between the two methods, it is difficult to determine which formulation has a longer sustained release period in soil, however, observations of the performance of the CMC hydrogel matrix used in this study during growth testing suggest that the sustained release period in soil is much longer than was observed in deionized water during the release study.

### Growth testing

Growth testing was used to determine if the RTDVs were effective in increasing the fertilizer use efficiency of the wheat plants compared to simply applying fertilizer dissolved in water. The experimental runs for each formulation of hydrogel were compared to each other by analysing the relative difference in the growth of the plants as compared to the controls rather than directly comparing the runs to each other in order to reduce the impact of extraneous factors such as day to day variability in humidity, light levels and temperature. Growths of the plants were compared using measurements of plant height and dry mass (at the end of the experiment). The focus of the study was primarily to reduce the amount of fertilizer used to promote plant growth, quantified using plant height, while maintaining plant yield, quantified using dry mass. The dry mass data gives a more complete measure of the plant growth. However, the fact that the plant must be uprooted in order to acquire a mass measurement makes the height measurement the best tool for determining the performance of the RTDVs over time as the plants grow. The plant height data gives a more complete picture of the effect of the hydrogels on the growth of the plants during the entire lifecycle, compared to the dry mass data, which can in turn be used to improve the release characteristics of the hydrogels to enhance performance.

**Figure 2:**
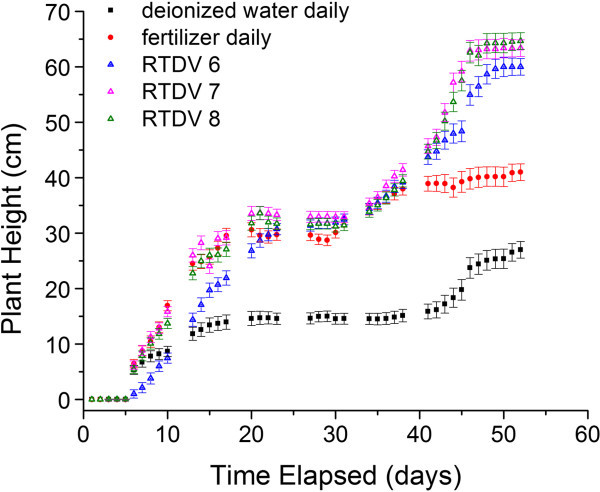
**The effect of drying RTDVs on the growth of wheat plants.** The plants were supplied with deionized water daily as a negative control (“black square symbol”), 20-20-20 fertilizer daily as a positive control (“red circle symbol”) or RTDVs at the start of the experiments. The RTDVs were loaded at 100% dose of 20-20-20 fertilizer and dried in the oven at 80°C for 0 h (“blue triangle symbol” RTDV 6), 24 h (“pink triangle symbol” RTDV 7), 48 h ("green triangle symbol" RTDV 8). Plant height (mean ± S.D., n = 3) is reported.

Fertilizing the plants daily over 50 days gives an effective dose of 2.15 g of fertilizer. RTDVs loaded with the same amount of 20-20-20 fertilizer as the total fertilizer given to the plants administered daily fertilizer doses. The results, show that the plants initially lagged behind those given daily applied fertilizer but from day 20 to day 43 the plants are approximately equal in height and after this point the plants given the RTDV outperform the plants fertilized daily in terms of growth (Figure 2, RTDV 6). All of the wheat plants reached the heading stage before the end of the experiment except for some of the plants given only deionized water, likely due to a lack of nutrients. After the experiment, the plants were uprooted and we observed that the hydrogels had a blue colouration from the residual fertilizer. These observations confirm that the release of fertilizer in soil is more sustained than the release observed in deionized water (Figure 1).

In addition to height data, dry mass measurements taken at the end of the growth experiment also show that the RTDVs were effective at increasing the growth and yield of the wheat plants. The data, shown in Table 2, indicates that the 100% dose hydrogels were able to increase the seed mass of the wheat plants almost double the amount produced by the plants receiving daily doses of fertilizer.

**Table 2:** **Mass measurements of wheat plants grown with root targeted delivery vehicles (RTDVs)**

Sample	Dry mass ± S.D. (%)	Dry seed mass ± S.D. (%)
Fertilizer applied daily	100.0 ± 20.8	100.0 ± 16.2
Deionized water daily	9.9 ± 2.4	11.6 ± 11.6
RTDV 4	20.6 ± 2.7	81.0 ± 6.8
RTDV 5	16.9 ± 0.0	66.5 ± 4.8
RTDV 6	78.9 ± 15.9	197.3 ± 63.7
RTDV 7	76.0 ± 2.8	198.9 ± 20.5
RTDV 8	69.5 ± 7.0	271.6 ± 32.4

### RTDV drying

In order to reduce burst release at the start of the growth period and increase release duration, we dried the hydrogels at 80°C for varying amounts of time. Drying the hydrogels before implantation resulted in significant improvement in the growth of the wheat plants. The plant heights for experiments performed with 100% dose RTDV subjected to 24 and 48 hour drying (Figure 2 RTDV 7, 8) show an increase in final plant height in the range of 3 to 5 cm for the plants with the hydrogels subjected to drying. Contrasted with the performance of the hydrogels without drying, the plants are consistently outpacing the plants given daily fertilizer in terms of height, rather than just in the last 10 days. In addition to increased plant height there was also a corresponding increase in the dry seed mass for the 48 hour drying of the hydrogels, as shown in Table 2. The RTDV with 48 hour drying had a 2.72 times increase in seed yield as compared to the daily fertilized plants, whereas the non-dried and 24 hour dried RTDV had a 1.97 and 1.99 times increase, over the daily fertilized plants.

There was significant improvement in seed yield using the RTDV compared to daily applied fertilizer. However, due to research indicating that the strength of the ionic bonds in the hydrogel is weakened by having a large concentration of ionic species (Rathna et al. 1996), additional methods of increasing the integrity of the hydrogels were investigated. Drying the hydrogel was introduced in order to attempt to minimize the amount of fertilizer released during the early growth period of the plants where the plants are using the nutrients contained in the seeds and instead provide nutrients more evenly over the course of the plant’s lifecycle to achieve higher growth yields (Sarkar et al. 2007, Yinbo et al. 1997).

**Figure 3:**
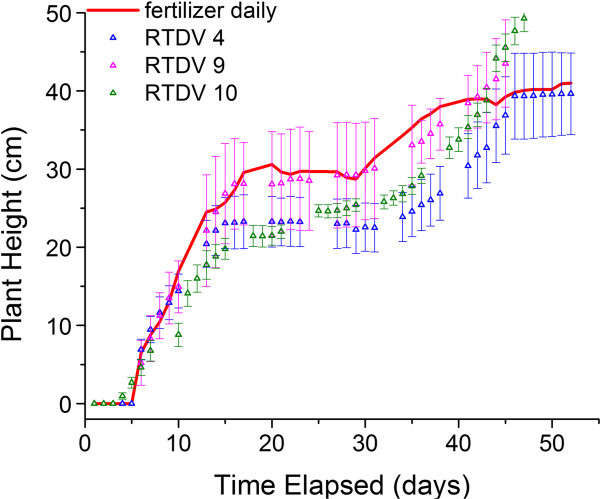
**The effect of loading dose and RTDV synthesis condition on the growth of wheat plants.** The plants were either supplied with 20-20-20 fertilizer daily as a positive control (“red horizontal line”) or RTDVs at the start of the experiments. The RTDVs were loaded with 20-20-20 fertilizer: 22% dose, 24 h drying (“blue triangle symbol” RTDV 4), 44% dose, 24 h drying (“pink triangle symbol” RTDV 9), 44% dose, heating during cross-linking (“green triangle sysmbol” RTDV 10). Plant height (mean ± S.D., n = 3) is reported.

An increase in plant height should correlate with an increase in mass, however, the mass data (Table 2) and height data (Figures 2 and 3) shows that although the plants grown with the device are taller than the fertilized plants, the mass of the fertilized plants is higher than the ones grown with the device. This discrepancy arose later in the plant growth cycle during the heading stage of plant development. In this stage the tillers (the grain bearing stalks) of the wheat grown with the RTDV grew much taller than those of the plants given daily fertilizer. This growth resulted in the height of the RTDV plants exceeding those for the fertilized plants despite a lower total mass for the RTDV wheat. Higher plant mass but lower seed yield can occur in cases of excessive nitrogen concentration (Oscarson, 2000). Based on these observations, it appears that the RTDV creates favourable conditions for development of grains on the plant, which corresponds to their higher seed yield, compared to the plants given daily fertilizer.

In order to reduce the reliance of the device on ionic bonding, drying of the devices was investigated as a method of increasing the strength of the RTDV. Research on CMC/polyvinylamine films showed that dried CMC films are able to form additional hydrogen bonds after already being bonded using ionic crosslinking (Feng and Pelton 2007). Drying the CMC hydrogel was intended to increase the bonding strength of the material through both the formation of these additional hydrogen bonds as well as increasing the time for water to fully hydrate the device.

### RTDV fertilizer loading variation

We performed growth experiments at varying degrees of fertilizer loading to test the limits of reduced fertilizer application before yield was adversely impacted. This is expected to yield an estimate of the improvement of nutrient absorption by the wheat. Growth experiments using RTDV 4 resulted in similar levels of growth to wheat plants given full daily doses of fertilizer (Figure 3). The lower level of fertilizer loading was able to maintain plant height and mostly maintain the seed mass of the plants but did not have the drastic improvement in growth seen with the 100% Dose RTDV. Table 2 shows that despite an approximate 78% reduction in the total amount of fertilizer delivered to the plants; yield only suffered by 19% for the 22% Dose RTDV 4 compared with an 89% reduction in the plants that did not receive fertilizer. Furthermore, assuming that the fertilizer released during the burst release phase could be recycled through the addition of a pre-washing step, it is possible that the actual dose delivered to the plant could be reduced by as much as 94% compared to the daily applied fertilizer by incorporating the reductions from both removing the burst release fertilizer and the initial loading of only a 22% dosage. The effectiveness of measures to remove the effect of the burst release will be evaluated in subsequent studies.

Growth experiments performed for RTDV 5, showed that increased drying time resulted in higher levels of plant height being achieved, however, seed yield and plant mass both decreased compared to the hydrogels dried for only 24 hours. Observations of the hydrogels that were removed from soil at the end of the experiment indicated that there was still residual fertilizer present in the RTDV, suggesting that the extended drying period of the RTDVs, coupled with the low loading of fertilizer, resulted in a release rate that was too slow to adequately supply the plants with nutrients.

RTDV 9 using a 44% fertilizer dose was tested using growth experiments in order to further quantify the effect of fertilizer loading on plant growth. The growth data (Figure 3) indicates that the 44% dose was effective at increasing growth in the wheat plants, according to plant height. The increase in fertilizer loading had diminishing returns according to the plant height data. Increasing the fertilizer loading from 22 to 44 to 100% dosage increased the growth each time, but the amount of growth gained decreased as the fertilizer level increased. This is likely due to the issue of the hydrogels being less able to effectively achieve sustained release at higher loading levels, as suggested by the release experiments and other studies (Rathna et al. 1996).

Based on the results of the previous release study and research showing the weakening effect of the high concentration of ionic compounds (Rathna et al. 1996), such as those present in the fertilizer, it was hypothesized that reducing the fertilizer loading in the hydrogel would increase the proportion of fertilizer released during the sustained release phase. The tests were performed at 22 and 44% dosage levels in the hydrogel in order to enable comparison with the previous release experiments and confirm the hypothesis that the lower fertilizer loading levels exhibited a more sustained release. 33% dose RTDVs were not tested due to the relative similarity between the release curves for the 33 and 44% release profile. These tests were also performed with varying levels of drying before implantation, as with the 100% dose RTDV.

Comparing the performance of the RTDV at different loading levels and drying times indicates that the drying process does improve the sustained release behaviour of the RTDV at higher loading levels. Drying the hydrogels for 48 hours resulted in a decrease in plant mass at 22% dosage but resulted in a large increase at 100%. Drying may be effective at mitigating the effect of higher fertilizer loads on the sustained release from the hydrogel, but the long drying time, cost of energy and risk of undersupplying nutrients to the plants make it undesirable for use in large scale production.

### Alternative RTDV synthesis methods

Each of these trials used RTDVs with 44% fertilizer dosage. The growth data of the hydrogels subjected to heated crosslinking (Figure 3) indicates that each of the different methods for increasing crosslinking density of the hydrogels were effective at increasing the growth of the wheat plants. Each method resulted in similar increases to plant height as 44% dose RTDVs that were subjected to the 24 hour drying time. The growth of the wheat plants in the trials for both alternative synthesis methods had more dramatic increases in growth during the latter half of the experiment compared to simple drying of the hydrogels. This fact suggests that these methods were more effective than drying at sustaining the release of the fertilizers from the hydrogels.

Alternatives to drying were investigated in order to attempt to avoid the large amount of synthesis time required for dried hydrogels and make the synthesis more economical. These alternatives centred on altering the synthesis process for the ionically crosslinked RTDVs in order to provide a more robust hydrogel that would be able to resist the weakening of the ionic bonding from the high fertilizer loads. The two alterations that were investigated were increasing the proportion of iron to calcium salts by increasing iron (II) and iron (III) chloride by 2 g and decreasing calcium chloride by 4 g per 400 ml crosslinking solution. Previous experiments suggested that increasing iron salt concentration in crosslinking CMC hydrogels resulted in a more heavily crosslinked structure. This is expected because the trivalent iron ions would increase ionic bonding strength between the CMC chains compared to divalent calcium ions. The previous experiments on CMC hydrogels were done without loading the hydrogel with any payload and resulted in hydrogels that were brittle and possessed a hard shell. The brittle morphology was not observed after the introduction of fertilizer as a payload which made this approach more viable. The second approach to increasing the hydrogel robustness involved subjecting the hydrogels to heating during the ionic crosslinking process. In this experiment, the hydrogels were heated to 40°C while crosslinking. The increase in temperature during crosslinking should increase the diffusion of salts into the hydrogel and result in a more densely and uniformly crosslinked hydrogel and a corresponding increase in sustained fertilizer release.

Overall, heating the hydrogels during crosslinking is the best alternative method due to differences in cost and time required compared to the altered crosslinking solution recipe or drying the hydrogels. Heating the hydrogels during crosslinking does not require the extra synthesis time that drying requires for improving the sustained fertilizer release of the hydrogels. In addition, the heated crosslinking hydrogels did not retain the colour of the fertilizer after the duration of the growth experiment, indicating a more complete release of fertilizer over the growth period of the plants compared to the dried hydrogels. This may indicate that the heating of the hydrogel serves to reduce both the amount of fertilizer that is loosely bound as well as the binding of the hydrogel shell phase, while increasing the proportion of fertilizer contained in the moderately crosslinked core phase, leading to a better sustained release profile. In comparison to the hydrogels synthesized with increased levels of iron salts, the heated crosslinking gels were more repeatable. In addition, it is unknown what effect changing the proportion of residual iron and calcium salts had on the growth of the wheat plants, it is possible that increasing the amount of iron, a potent micronutrient for many plants, delivered by the hydrogel served to mitigate excess salinity that may have been introduced by the calcium salts or fertilizer (Delgado and Sanchez-Raya 2007).

### RTDV degradation testing

**Figure 4:**
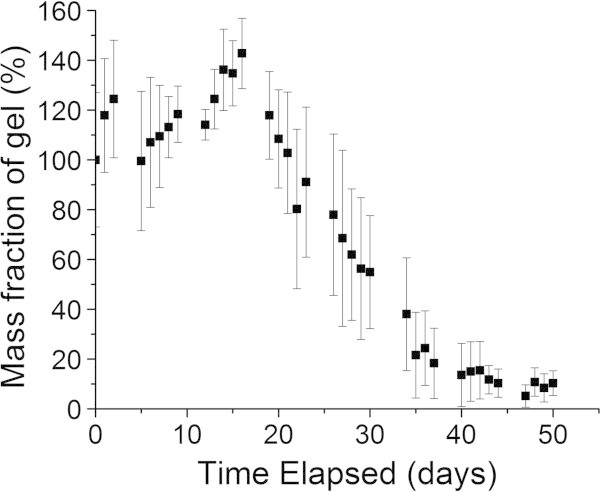
**Degradation of RTDV in soil.** RTDV 1 loaded with 22% dose of 20-20-20 fertilizer was placed in the soil and supplied with deionized water daily. The mass (mean ± S.D., n = 3) of RTDV has been normalized against the initial mass.

RTDVs were left in soil and regularly exhumed and weighed to gauge the amount of mass loss as a result of degradation of the gel. The results of these trials (Figure 4) show an initial increase in hydrogel mass in the first 15 days due to water absorption, followed by steady degradation of the hydrogel. Even with a large amount of water exposure, the hydrogels are able to avoid complete degradation despite being subjected to 50 watering cycles, ending with approximately 10% of their original mass. In addition, the remaining hydrogel was observed after this time period to have retained a small amount of the encapsulated fertilizer due to the presence of the 20-20-20 fertilizer’s blue colouration in the hydrogel. This experiment suggests that the hydrogel would be able to survive and continue releasing fertilizer for an extended period of time in soil but would be removed from the soil by degradation after the end of the growing season once they are no longer needed. Although separate degradation tests for dried hydrogels were not performed, it was observed during the growth experiments that the dried hydrogels were much more resistant to degradation over the course of these experiments than non-dried hydrogels. Hydrogels watered on a weekly basis had much less loss of mass compared to those watered on a daily basis over the same time period. Some of the mass loss observed is a result of the initial loading of water in the hydrogel being diffused out of the hydrogel by the drier soil over time. These hydrogels slowly degraded over the 50 day period, but retained much of their initial mass. The weekly watered hydrogels were able to retain approximately 89% of their original mass (not including absorbed water).

In order to be effective as a controlled release fertilizer agent, the RTDVs should remain intact in soil over the course of the growing season. Optimally the RTDVs will only require insertion into soil during crop planting with no need for reapplication until the next growing season, but will be not remain in the soil once no longer needed. In order to investigate the RTDVs ability to remain intact for this length of time, tests in which RTDV with 22% fertilizer dosage and no drying were placed into soil and subjected to conditions similar to those experienced by the hydrogel during the growth experiments. However, wheat plants were not grown along with these gels, instead the hydrogel mass was monitored to give an indication of the amount of physical degradation of the RTDV.

This indicates that the RTDVs are likely a viable method for fertilizer release over the course of an entire growing season in field conditions. The observed oscillation of the hydrogel mass in time with the watering cycle also suggests that the RTDVs may be effective at storing water in soil over periods of drought, as it takes approximately 27 days of weekly watering before the hydrogel reaches a minimum mass. Even though the hydrogels were able to repeatedly absorb and release water, significant changes in the volume of the RTDVs were not observed, this is advantageous since changing the height of soil and disturbing root systems is undesirable during plant growth. The resistance of the dried RTDVs to degradation could be an issue with regard to a build-up of litter in fields if the hydrogels do not effectively biodegrade after the growing season. However, combined with their lengthened release time, the dried RTDVs may be viable for release over multiple seasons if they are able to withstand being in soil over the winter months. The ability of the RTDVs to act as a medium for improving drought resistance of crops will be explored in subsequent investigations.

## Conclusions

Overall, the RTDVs are a promising platform for increasing the fertilizer use efficiency of agricultural crops. The RTDV maintained seed yield of crops despite reductions of fertilizer use of up to 78%, with the possibility that this reduction could be increased to as much as 94% with further optimization. The difference in the total plant mass and seed mass observed may also indicate that the CRF was altering the nutrient availability in the soil. In addition, there is potential for improving the performance of the RTDVs further in order to maximize the efficient use of fertilizer and allow larger reductions in fertilizer use in order to help reduce costs and environmental issues caused by fertilizer pollution. Alternative uses for the RTDVs in terms of drought mitigation were also identified as possible avenues of investigation. However, further investigation will be required in order to fully explore the ability of the RTDVs in increasing fertilizer use efficiency in a wider variety of crop plants and field conditions.

## Abbreviations

(CMC): Carboxymethyl cellulose
(CRF): Controlled release fertilizer
(RTDV): Root targeted delivery vehicle.
